# Retraction: Neuroprotective effect of sodium alginate against chromium-induced brain damage in rats

**DOI:** 10.1371/journal.pone.0343068

**Published:** 2026-02-18

**Authors:** The PLOS One Editors

After this article [1] was published, concerns were raised about the comet assay in Fig 5.

Specifically, in Fig 5B, regions of the central and left-hand comet tails (the fragmented DNA signal) appear similar to each other. Upon editorial follow-up, the corresponding author GMH stated that DNA migration close to the nucleus often appears similar among comets with comparable levels of DNA damage.

Corresponding author GMH stated that the quantitative data underlying all figures are no longer available, but they provided the underlying comet assay images. However, after editorial review, the *PLOS One* Editors consider that the underlying image data provided do not resolve the issue and raise further concerns about the integrity and reliability of these experimental data.

In light of the above issues, which raise concerns about the reliability and integrity of the reported results, the *PLOS One* Editors retract this article.

GMH and REH did not agree with the retraction. EMS either could not be reached or did not respond directly.
